# Low Bone Turnover and Decreased Bone Matrix Mineralization at Diagnosis in Children with Acute Lymphoblastic Leukemia

**DOI:** 10.1007/s00223-026-01509-7

**Published:** 2026-04-02

**Authors:** Pauliina Utriainen, Markus A. Hartmann, Stéphane Blouin, Takolander Sofia, Outi Mäkitie, Nadja Fratzl-Zelman

**Affiliations:** 1 Division of Pediatric Hematology and Oncology and Stem Cell Transplantation, Children’s Hospital, University of Helsinki and Helsinki University Hospital, HUS, Stenbäckinkatu 9, 00029 Helsinki, Finland; 2 Research Program for Clinical and Molecular Metabolism, University of Helsinki, Helsinki, Finland; 3 Ludwig Boltzmann Institute of Osteology at the Hanusch Hospital of OEGK and AUVA Trauma Centre Meidling, 1st Medical Department Hanusch Hospital, Vienna, Austria; 4 Vienna Bone and Growth Center, Vienna, Austria; 5 Folkhälsan Research Center, Genetics Research Program, Helsinki, Finland; 6 Department of Molecular Medicine and Surgery, Karolinska Institute, and Clinical Genetics, Karolinska University Hospital, Stockholm, Sweden

**Keywords:** Acute lymphoblastic leukemia, Skeletal complication, Bone mineral density (BMD), Bone mineralization density distribution (BMDD), Bone histomorphometry, Quantitative backscattered electron imaging (qBEI)

## Abstract

In children with acute lymphoblastic leukemia (ALL), skeletal complications are well documented, but their exact origin remains unclear. In the present study we investigated bone mineralization density distribution by quantitative back-scattered electron imaging (qBEI) and bone histomorphometry by light microscopy in pediatric ALL patients at diagnosis, before any cancer treatment. In our one-center LELU study, we obtained bone marrow biopsies from the posterior iliac crest in 11 boys and 15 girls (median age 4.3; range 2.6–15.8 years) in connection with ALL diagnostic sampling. Blood samples were drawn for biochemistry and BMD was measured by DXA. Despite normal DXA-derived BMD, qBEI revealed undermineralization of bone matrix in our ALL-patient cohort. The average degree of mineralization was reduced (− 8%) and the proportion of lowly mineralized bone area was increased compared to pediatric reference values (+ 152%, both *p* < 0.0001). Bone histomorphometry revealed normal osteoid thickness, but markedly reduced osteoblast and eroded surface. Bone marrow fibrosis was observed in 24/26 samples. Neither BMD nor serum 25-OH-vitamin D correlated with bone matrix mineralization or histomorphometric parameters. In conclusion, our study showed a marked hypomineralization of the bone matrix in children at ALL diagnosis, prior to cancer treatment. These data suggest an early and significant impact of the ALL-disease process on bone homeostasis and material quality.

## Introduction

The survival rate of pediatric acute lymphoblastic leukemia (ALL) patients has continuously improved, exceeding 90% today [1, 2]. In the context of excellent prognosis, the long-term adverse effects of the disease and its treatment have become greatly important.

Skeletal complications, including low bone mineral density (BMD), vertebral compression fractures, nonvertebral low-trauma fractures and osteonecrosis are common in childhood ALL [3, 4]. In children, vertebral fracture is always a sign of severe skeletal morbidity. In a large Canadian study (STOPP consortium), vertebral fractures were found in 16% of children at ALL diagnosis [4], and their cumulative incidence within 6 years was increased to 32.5% when systematically screened by MRI [5]. The best predictors for developing vertebral fractures in the follow up included: a vertebral fracture already at diagnosis, low baseline BMD and greater glucocorticoid exposure [5]. These data are in line with studies reporting that at diagnosis, 13–40% of patients with ALL have low BMD or osteopenia [6–8]. During subsequent treatment, BMD further decreases [9–11] resulting in a six-fold increase in fracture incidence in ALL patients during therapy compared with healthy controls [9].

Symptomatic osteonecrosis (ON) develops in approximately 2–10% of children with ALL [3], the risk being highest among teenagers [12–15]. Other risk factors include intense glucocorticoid administration [14, 15], female sex [12–14] and overweight [14]. Prevalence of ON is higher if systematically screened. In the ongoing OPAL trial, MRI screening revealed ON lesions in 9.2% of children with ALL already at leukemia diagnosis [16], most being non-symptomatic. In line with these observations, the STOPP consortium reported a progressive decrease in BMD during leukemia treatment, and there was an association between the reduction of BMD and number of ON lesions observed by MRI after the treatment [17].

Although the skeletal complications in ALL are well acknowledged, their underlying tissue-level mechanisms are not well understood [11, 18]. It is unclear to what extent skeletal morbidity can manifest already at diagnosis before initiating leukemia treatment. This is of particular interest because in some cases, skeletal complications are seen already at diagnosis, before any cancer treatment [4, 19].

Bone histomorphometry and quantitative backscattered electron imaging (qBEI) are powerful methods to evaluate bone tissue characteristics at the microscopic level – including bone cell metabolism and the degree of mineralization of the bone matrix [20]. To better understand the pathomechanisms of skeletal complications in childhood ALL, we set to characterize bone turnover and mineralization pattern by qBEI in bone biopsy samples obtained from posterior iliac crest in newly diagnosed pediatric patients with ALL. qBEI findings were correlated with clinical presentation, routine blood analysis and BMD measurements.

## Material and Methods

### Study Design and Sampling

**Fig. 1:**
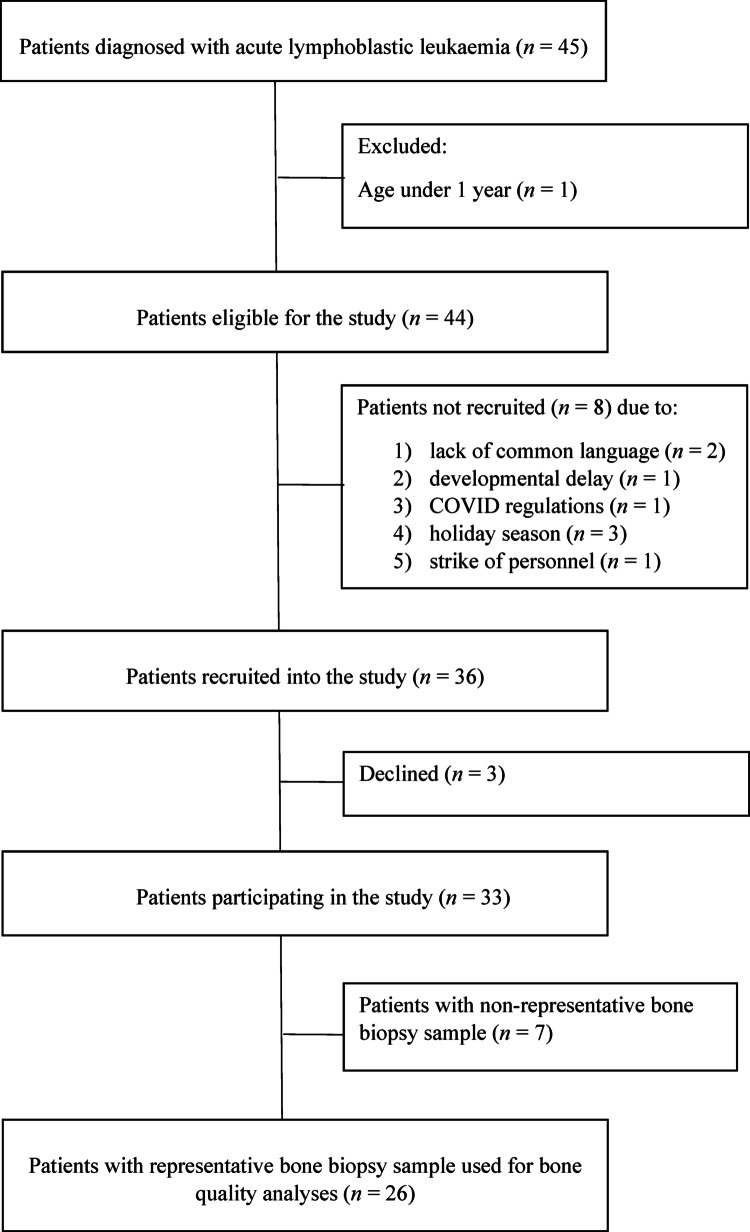
Flow chart

This cross-sectional one-center LELU study included 26 patients with ALL. Altogether 45 patients aged < 16 years were diagnosed with ALL at the Children’s Hospital, Helsinki University Hospital, Finland, between June 2019 and May 2022. Exclusion criteria included age < 1 year and Down syndrome. Among the 44 eligible patients (pre-B-ALL, N = 41; T-ALL, N = 3), eight patients were not recruited due to lack of common language, developmental delay, COVID19 regulations, holiday season or strike of the personnel. Altogether 33 of the 36 patients invited consented to participate. Representative biopsy samples for bone marrow and mineralized tissue evaluation were obtained from 26/33 ALL patients (11 males, 15 females) (Flow chart, Fig. 1).

All participants were Caucasians with no previous skeletal, endocrine or malignant diseases.

A written informed consent was received from the guardians of all patients; participants aged > 6 years also gave their own written assent. The study was approved by the Research Ethics Committee of Helsinki University Hospital.

Prior to cancer treatment, bone marrow biopsy samples were obtained from the posterior iliac crest in connection with routine ALL diagnostic sampling with a trephine (Jamshidi™ Bone Marrow Biopsy Needle, CareFusion; or T-Lok™ Bone Marrow Biopsy Needle, Argon Medical Devices, Inc.) having an inner diameter of 11G and 13G corresponding to 2.388 mm and 1.803 mm, respectively. For the present study, we obtained an additional bone biopsy sample after the diagnostic sample.

At ALL diagnosis, a blood sample was taken for analysis of parameters of vitamin D and calcium metabolism. Plasma calcium, ionized calcium, alkaline phosphatase (ALP) and creatinine were measured using standard methods at the University Hospital’s core laboratory HUSLAB. Serum 25-hydroxy-vitamin D (25-OHD) was assayed by high-performance liquid chromatography [21]. Parathyroid hormone (PTH) was measured with immunoassay based on chemiluminescence (either Abbott Architect or Siemens Atellica) with limit of detection 0.53 pmol/l and 0.64 pmol/l, intra-assay CV 6.1% and 4.6% and inter-assay CV 6.4% and 7.8%, respectively.

Clinical details concerning bone health, as well as patient and leukemia characteristics were collected from hospital records.

### Bone Mineral Density (BMD)

To evaluate BMD, dual-energy x-ray absorptiometry (DXA) analysis was performed within 4 weeks of diagnosis. The areal BMD (g/cm^2^) was assessed for total body, lumbar spine (L1-4 vertebrae, LS) and for femoral neck (FN) with DXA (Hologic Discovery A, Paediatric software, version 13.5.1. or Hologic Horizon A, Pediatric software version 13.6.1.1; Hologic, Marlborough, MA). The DXA measurements were converted to *z*-scores using equipment specific age- and sex-adjusted reference data for Caucasian American children. These references have been validated for Finnish children [22]. The obtained *z*-scores were used for the statistical analyses.

### Bone Histomorphometry and Bone Mineralization Density Distribution

Bone specimens were fixed in 70% ethanol, dehydrated in graded series of alcohol and embedded in polymethylmethacrylate. Static histomorphometric parameters of bone remodeling were evaluated in 3 µm thick sections stained with Goldner trichrome using a light microscope (Axiophot, Zeiss, Oberkochen, Germany) equipped with a digital camera (AxioCam HRc, Zeiss, Germany). Indices of bone formation and resorption were evaluated and compared to published pediatric reference values [23, 24]. Structural parameters could not be assessed, as the size of the samples did not allow for representative measurements.

The residual sample blocks were prepared for qBEI using standard procedures [25–27]. The surfaces were scanned with a field emission scanning electron microscope (FE-SEM SUPRA 40, Zeiss, Oberkochen, Germany) equipped with a 4-quadrant solid state backscattered detector. The grey levels reflecting the local calcium content were calibrated with standards made of carbon and aluminum. Images were taken with a spatial resolution of 1.76 μm per pixel. From the obtained images, grey level histograms reflecting the bone mineralization density distribution (BMDD) were assessed and characterized by the following parameters: the mean calcium concentration (CaMean, weight% calcium), the most frequent calcium concentration (CaPeak, weight% calcium), the full width at half maximum of the distribution (CaWidth, Δ weight% calcium) as an indicator for heterogeneity of mineralization, as well as CaLow and CaHigh that measure the fraction of lowly and highly mineralized bone area (% bone area), respectively and compared to pediatric reference values [27].

### Statistical Analyses

BMDD results are reported as median [25th, 75th interquartile range] or mean (± SD). Shapiro–Wilk test was used to test for normality of distributions. Normality was assumed if p > 0.05. For normally distributed data, unpaired *t*-test was conducted. If normality was violated a Mann–Whitney test was performed to compare the means between the groups. Since histomorphometric references were only available as mean (SD), unpaired t-tests were conducted regardless of normality of data. For subgroup analyses Mann–Whitney test was used because of small group sizes. Pearson and Spearman’s correlation tests were used to analyze correlations between parameters in ALL patients. In all cases, significance was considered at p < 0.05. Statistical analyses were carried out using GraphPad Prism 10.0.3, Python Scipy 1.11.4 and IBM SPSS Statistics (Version 29.0.0.0(241)).

## Results

### Clinical Characteristics

The patients´ characteristics and clinical bone metabolic parameters are compiled in Table 1.

**Table 1:** Clinical characteristics of the 26 pediatric patients (all Caucasians) with acute lymphoblastic leukemia (ALL)

	Total N	Percentage	
Diagnosis (B-ALL/T-ALL)	24/2	92.3/7.7	
Sex (female/male)	15/11	57.7/42.3	
	Median	Range	IQR
Age (years)	4.3	2.6 to 15.8	3.5 to 7.2
Height z-score	+ 0.18	− 1.95 – + 2.53	− 0.51 – + 0.71
ISO-BMI (kg/m2)	20.4	14.3 to 31.7	18.4 to 24.5
B-Leuk (E9/L)	6.1	1.3 to 550.4	2.8 to 28.5
P-Ca (mmol/L)	2.32	2.04 to 2.65	2.23 to 2.45
P-Ca-ion (mmol/L)	1.24	1.16 to 1.90	1.22 to 1.27
P-Pi (mmol/L)	1.80	1.05 to 2.71	1.51 to 1.95
P-ALP (U/L)	135	64 to 287	105 to 191
S-25-OHD (nmol/L)	58	34 to 120	49 to 67
P-PTH (ng/L)	28	4.6 to 110	15 to 64
BMD LS z-score	− 0.5	− 2.60 to + 2.30	− 1.3 to + 0.4
BMD Total body z-score	0.15	− 1.1 to + 2.7	− 0.23 to + 1.2

ALL, acute lymphoblastic leukemia; B, whole blood; P, plasma; ALP, alkaline phosphatase; S, Serum; PTH, parathyroid hormone; IQR, interquartile range; BMD, bone mineral density; LS, lumbar spine

### Leukemia Characteristics

#### Blood Count

Leukocyte count varied between 1.3 × 10^9^ and 550 × 10^9^ per liter. Altogether three patients had leukocyte count > 50 × 10^9^ per liter, which is considered high-risk feature in concurrent ALL protocols. One of these patients harbored T cell ALL. Blood count features are shown in Table 2.

#### Bone Marrow Infiltration

All patients showed bone marrow infiltration by leukemic lymphoblasts.

According to pathologist’s report, 95% to 100% of the visible cells were leukemic blasts in all but one patient. In the remaining one patient, approximately 50% of the cells in the biopsy sample were blasts. In the bone marrow aspiration samples, leukemic blast cells comprised 49–98% of blood cells, as measured with flow cytometry.

#### Cytogenetic Aberrations

Genetic characteristics of the ALL clones in each patient are shown in Table 2. Altogether 17 patients had favorable-risk genetics (hyperdiploid chromosomes N = 8 and *ETV6::RUNX1* (*TEL::AML*) gene fusion N = 9) in their leukemic clones, while 3 patients harbored poor-risk genotype (*iAMP21*; *KMT2A*; *NRAS* with mixed phenotype) (Table 2).

#### High-Risk Features of ALL

When considering the clinical, hematological and genetic factors associated with poor prognosis, eight patients (31%) fulfilled at least one high-risk criterion (age > 10, leukocyte count > 50 × 10^9^ per liter, unfavorable genetics, T-cell immunophenotype; Table 2).

### Skeletal Complications at Diagnosis

Patients with ALL were not routinely screened with radiographs for skeletal complications at diagnosis. Among the 26 ALL patients, two boys originally presented with skeletal symptoms assessed by MRI. One of them, a teenage boy, presented with back pain and multiple vertebral compression fractures. His BMD *z*-scores were + 0.1, -0.7 and -1.6 for total body, LS and FN, respectively. Another patient (a 3-year-old boy) had multifocal osteonecrosis in lower limbs. His ALL manifested with fever and limping—and the leukemia was first suspected due to the abnormal BM signal in the MRI.

### Calcium and Vitamin D Metabolism

At diagnosis, one patient had asymptomatic hypocalcemia and another asymptomatic hypercalcemia together with low PTH level. Two patients had high and 6 low PTH levels. High PTH levels were not associated with low 25-OHD. Altogether 6/25 patients (25%) had vitamin D insufficiency (25-OHD < 50 nmol/L). The majority (N = 14; 58%) had 25-OHD between 50 and 80 nmol/L.

### Bone Histomorphometry

**Fig. 2:**
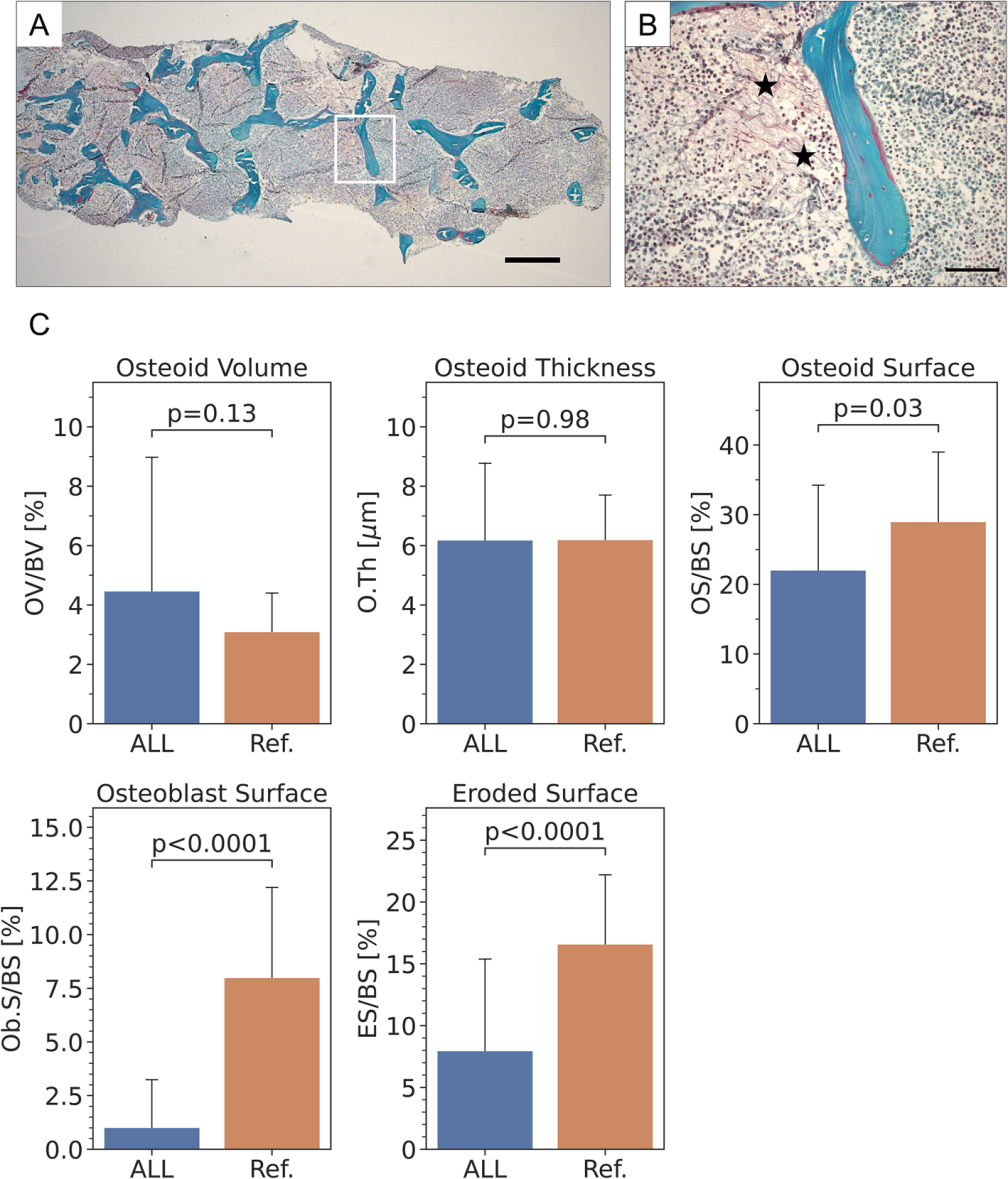
Results of bone biopsy sample histomorphometric analysis from a child with ALL (boy, 7-year-old at biopsy): **A** and **B** Goldner stained histological section shows mineralized bone in green–blue and unmineralized matrix (osteoid) in red. **A** overview of the sample. Little osteoid is viewed. Scalebar: 500 µm. The area within the white square is shown at higher magnification in B. **B** Detail from A: Trabecular feature shows mostly mineralized matrix with thin osteoid seams. Scalebar: 100 µm. Some areas with fibrosis can be observed (black asterisks). **C** Results from histomorphometric analysis for all patients (n = 26): Shown static bone formation and resorption parameters are Osteoid Volume, Osteoid Thickness, Osteoid Surface and Osteoblast Surface as well as Eroded Surface as a surrogate of osteoclastic activity. Bars show data as mean (SD). *p*-values given stem from unpaired t-test comparisons with healthy controls from [23, 24] and reported as mean (SD)

Representative light-microscopy images of a Goldner stained section from a biopsy sample obtained from an ALL patient are shown in Fig. 2A-B; histomorphometric results are compiled in Table 3. While osteoid volume and osteoid thickness in ALL patients did not significantly differ from control values (all *p* > 0.05), osteoid surface, osteoblast surface and eroded surface were significantly lower (*p* = 0.03, *p* < 0.0001 and *p* < 0.0001, respectively, Fig. 2C). Osteoid surface was decreased by 24% in ALL patients, whereas osteoblast surface and eroded surface were reduced by − 88% and − 52%, respectively. We observed bone marrow fibrosis in all except two samples (both pre-B ALL); fibrosis was pronounced in 12/26 samples (Fig. 2B).

To evaluate whether the extent of leukemic infiltration in the bone marrow influenced parameters of bone formation, the cohort was divided into those with leukemic blast count ≥ 90% by flow cytometry (N = 14) and those with blast count < 90% (N = 12). There were no significant differences in the bone formation markers by histomorphometry between these subgroups (*p* > 0.4 for all). Bone histomorphometric parameters were also similar between those with a good-prognosis cytogenetics (N = 17) and those without (N = 9) (*p* > 0.24 for all). When comparing patients fulfilling any high-risk criterion (Table 2; N = 8) to others, those with high-risk features showed lower eroded surface percentage (median 2.4% vs 7.5%; *p* = 0.026).

**Table 2:** Patient and disease characteristics in 26 pediatric patients with newly diagnosed acute lymphoblastic leukemia, in whom a bone biopsy sample was examined by histomorphometry and quantitative Backscattered Electron Imaging (qBEI)

Patient	Sex	High-risk feature at diagnosis	Age (years)	Leukocyte count at diagnosis (× 10^9^)	Immuno-phenotype	Genetic aberrations
No 1	M	No	< 10	2.7	B-ALL	*TEL::AML1* (*ETV6::RUNX1*) gene fusion
No 2	F	No	< 10	13.8	B-ALL	Hyperdiploid
No 3	F	No	< 10	6.0	B-ALL	Amplification of chromosome 21
No 4	M	No	< 10	2.2	B-ALL	Hyperdiploid
No 5	F	Yes (genetics)	< 10	30.4	B-ALL	*NRAS* mutation
No 6	M	Yes (age; genetics; leukocyte)	> 10	247.1	B-ALL	*KMT2A* mutation
No 7	F	No	< 10	1.3	B-ALL	Amplification of *HLF* and *RUNX1* genes
No 8	F	No	< 10	43.5	B-ALL	*TEL::AML1* (*ETV6::RUNX1*) gene fusion
No 9	F	No	< 10	3.6	B-ALL	*TEL::AML1* (*ETV6::RUNX1*) gene fusion
No 10	M	Yes (age)	> 10	42.5	B-ALL	*TCF3::PBX1* fusion
No 11	F	No	< 10	26.5	B-ALL	*TEL::AML1* (*ETV6::RUNX1*) gene fusion
No 12	M	No	< 10	6.1	B-ALL	*TEL::AML1* (*ETV6::RUNX1*) gene fusion
No 13	F	No	< 10	2.6	B-ALL	Amplification of PBX1, ABL2, HLF and RUNX1 genes
No 14	F	No	< 10	2.9	B-ALL	Gain of *HLF* and *RUNX1* genes
No 15	M	Yes (T cell; leukocytes)	< 10	550.4	T-ALL	17q11.2 (NF1 gene) copy number loss
No 16	M	No	< 10	7.2	B-ALL	Hyperdiploid
No 17	M	Yes (age; genetics)	> 10	1.3	B-ALL	iAMP21*; deletion of *ETV6* and *IKZF1* genes
No 18	M	Yes (leukocytes)	< 10	146.2	B-ALL	Hyperdiploid
No 19	F	Yes (age)	> 10	2.2	B-ALL	*TEL::AML1* (*ETV6::RUNX1*) gene fusion
No 20	F	No	< 10	12.8	B-ALL	*TEL::AML1* (*ETV6::RUNX1*) gene fusion
No 21	F	No	< 10	7.6	B-ALL	*TEL::AML1* (*ETV6::RUNX1*) gene fusion
No 22	M	Yes (T cell)	< 10	4.8	T-ALL	*CDKN2A/B* deletion
No 23	M	No	< 10	1.3	B-ALL	Hyperdiploid
No 24	F	No	< 10	5.3	B-ALL	Hyperdiploid
No 25	F	No	< 10	6.4	B-ALL	*TEL::AML1* (*ETV6::RUNX1*) gene fusion
No 26	F	No	< 10	5.4	B-ALL	Amplification of *MLL*, *ETV6* and *RUNX1* genes

iAMP21, intrachromosomal amplification of chromosome 21

*Hyperdiploid = high hyperdiploid chromosome number, between 51 and 67

ALL, acute lymphoblastic leukemia; F, female; M, male

**Table 3:** Bone histomorphometry and bone mineral density distribution, measured by quantitative Backscattered Electron Imaging, at cancer diagnosis in 26 pediatric patients with acute lymphoblastic leukemia in comparison with reference data

Results of biopsy samples analyses			
Parameters	References*	ALL	*p* value
Bone Histomorphometry			
Osteoid thickness (µm)	6.2 (1.5)	6.2 (2.6)	0.98
Osteoid surface/bone surface (%)	29 (10.0)	22.3 (11.6)	0.03
Osteoid volume/bone volume (%)	3.1 (1.3)	4.5 (4.5)	0.13
Osteoblast surface/ bone surface (%)	8.0 (4.2)	1.0 (2.2)	< 0.0001
Eroded surface/ bone surface (%)	16.6 (5.6)	8.0 (7.4)	< 0.0001
Quantitative Backscattered Electron Imaging (qBEI)			
CaMean (weight % calcium)	22.5 (0.7)	20.6 (0.9)	< 0.0001
CaPeak (weight % calcium)	23.4 (0.7)	21.8 (0.9)	< 0.0001
CaWidth (Δ weight % calcium)	3.6 [3.5; 3.9]	4.7 [4.5; 5.0]	< 0.0001
CaLow (% bone area)	5.57 [4.8; 6.7]	13.8 [10.3;21.9]	< 0.0001
CaHigh (% bone area)	1.5 [0.6; 2.2]	1.8 [0.6; 2.8]	0.68

Mean (SD) or median with IQ [25th, 75th] are shown

*Reference data for healthy children were reported in [23] for static parameters of bone formation (O.Th, OS/BS, OV/BV and Ob.S/BS) as well as in Glorieux FH et al. [24] for static parameters of bone resorption ES/BS. As no single results were available from these data, mean (SD) were used to perform unpaired t-tests in bone histomorphometry irrespective of the normality of the data. Mähr M et al. [27] report reference values for quantitative backscattered electron imaging. Data are presented as mean (SD) or median [IQR] as appropriate

### Bone Mineralization Density Distribution (BMDD)

**Fig. 3:**
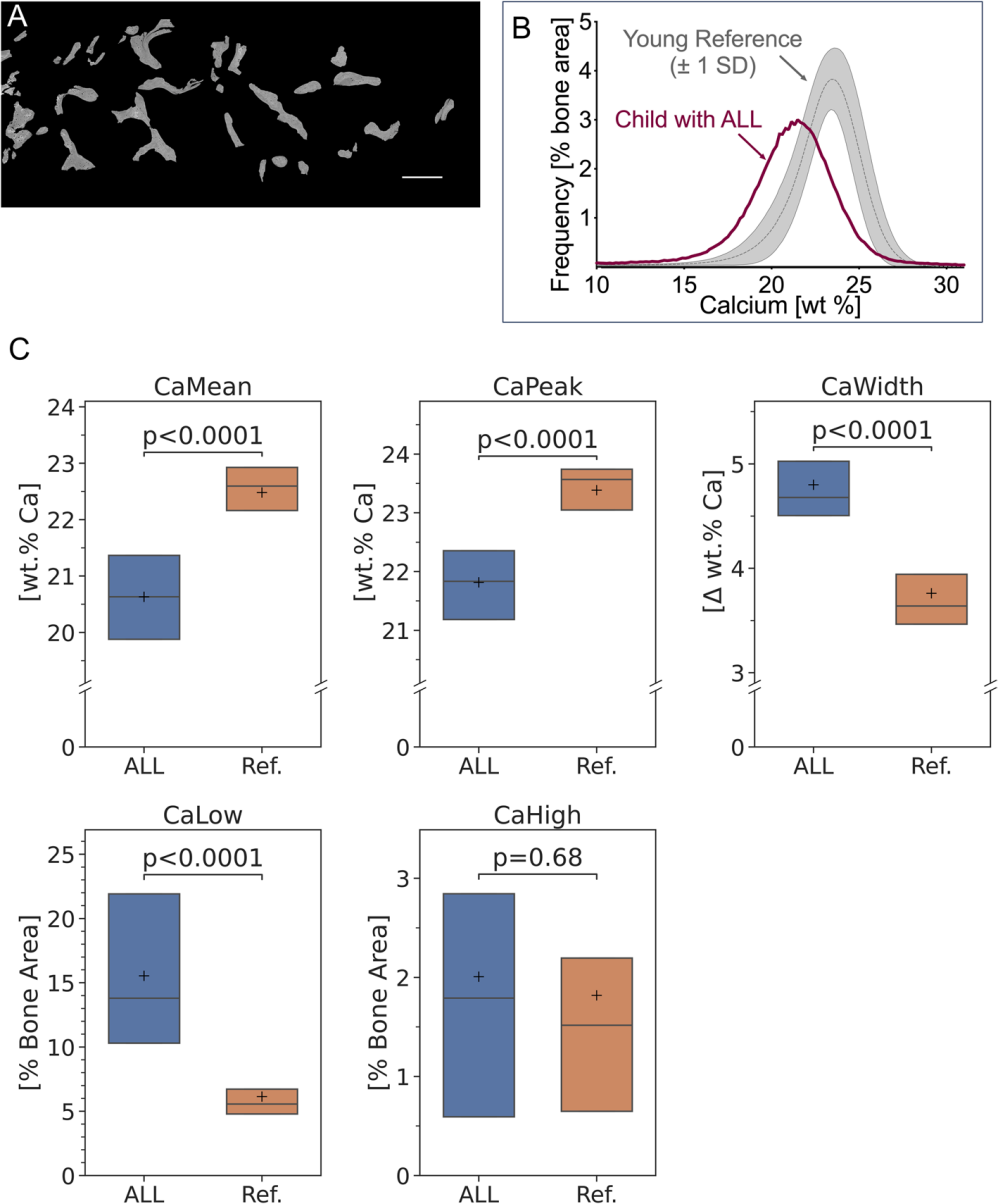
**A** Quantitative backscattered electron microscopy image of the sectioned sample from the same patient with ALL (boy, 7-year-old at biopsy) as in Fig. 2: mineralized matrix is shown as a shade of gray, unmineralized matrix, bone marrow and embedding medium appear black. Scalebar: 500 µm. **B** Corresponding Bone Mineralization Density Distribution (BMDD) from the sample shown in Fig. 1A. The BMDD curve of the child with ALL is shifted to the left, i.e., towards lower bone matrix mineralization compared to the reference cohort of healthy children published previously [27]. **C** Comparison of the following BMDD parameters with healthy references: the mean calcium content (CaMean), the most frequent calcium content (CaPeak), the heterogeneity of mineralization measured by the width of the BMDD at half maximum (CaWidth) as well as the amount of lowly and highly mineralized bone area (CaLow and CaHigh). The horizontal line shows the median, while the cross corresponds to the mean of data. Box size equals the interquartile range (IQR). P-values given stem from unpaired *t*-tests or Mann–Whitney comparisons between the groups, as appropriate

Figure 3 shows a representative overview image obtained by qBEI and Fig. 3B depicts the corresponding BMDD curve. In comparison to the reference BMDD of healthy children, patients with ALL exhibited significant hypomineralization of the bone matrix, as indicated by a curve shifted toward lower calcium concentrations (Fig. 3C, Table 3). Correspondingly, both the mean calcium content and the most frequent calcium concentration were reduced (CaMean: -8%, CaPeak: -7%, both *p* < 0.0001). Additionally, there was an increase in the proportion of lowly mineralized bone and greater heterogeneity in mineralization (CaLow: + 153%, CaWidth: + 28%, both *p* < 0.0001). The proportion of highly mineralized bone area (CaHigh) did not differ from the reference group (*p* = 0.68).

Compared to females, males had lower CaMean (− 2.4%, *p* = 0.04) and CaPeak (− 3.1%, *p* = 0.02), but higher CaLow (+ 28.5%, *p* = 0.02).

There were no differences in the mineralization parameters between the bone marrow blast count subgroups (≥ 90% vs. < 90%) or between those with favorable genetics and others (*p* > 0.2 for all). A trend towards higher percentage of lowly mineralized bone in those with high leukocyte count at diagnosis (N = 3) compared with others was seen (median 24% vs. 13%, *P* = 0.052).

DXA-derived BMD *z*-score at LS associated with lower eroded surface (Spearman’s correlation − 0.495; *p* = 0.023) and total body BMD *z*-score with lower osteoid surface but no other significant associations were found between BMD *z*-scores and BMDD parameters.

Neither bone histomorphometric nor BMDD parameters were associated with circulating 25-OHD, calcium, ALP or PTH (all NS, data not shown).

The teenage male patient with serial vertebral fractures at diagnosis presented with very low eroded surface (2.6%), osteoid volume (0.41%), osteoid surface (11.3%) and osteoblast surface (0%), while the 3-year-old boy with multifocal osteonecrosis also had very low eroded surface (1.1%) and no observable osteoblasts (0%).

## Discussion

This study examined 26 newly diagnosed pediatric patients with ALL for bone tissue characteristics, using iliac bone biopsy samples obtained in connection with routine diagnostic bone marrow sampling. All patients showed bone marrow infiltration by leukemic lymphoblasts, low osteoblast indices by bone histomorphometry and low bone matrix mineralization by qBEI.

Several studies have shown that BMD decreases during ALL treatment [3, 9–11]. BMD deficits and skeletal complications in ALL have traditionally been associated with the leukemia treatment, particularly the administration of glucocorticoids. More intensive glucocorticoid dosing increases the risk for osteonecrosis [3, 15]. Nonetheless, it is also known that BMD can be abnormally low already at ALL diagnosis—and we and others have reported on skeletal complications at diagnosis, including osteonecrosis [19] and vertebral fractures [4, 28, 29], that are highly prevalent also in non-symptomatic patients [4, 16].

Our histomorphometric analyses revealed reduced osteoblast and eroded surface, a marker of osteoclast activity, indicating low bone turnover in our ALL-patient cohort at diagnosis. Interestingly, osteoid indices were within normal range or slightly reduced. Thus, there were no signs of osteomalacia, i.e. mineralization defects on bone histology [30]. These findings are consistent with a previous report assessing bone biopsy samples in 23 children with ALL [18] that found no differences in total osteoid indices, but a decrease in osteoblast and osteoclast number and in “active osteoid area” compared to children with other cancers. Also in line with the current results, our recent study with Swedish collaborators showed decreased circulating levels of bone formation and resorption markers at leukemia diagnosis [31]. Previous studies that have measured circulating bone turnover markers in pediatric ALL patients at diagnosis [9, 11, 32–34], reported similarly decreased bone formation markers and some also decreased bone resorption markers [32, 34]. Thus, our current study confirms, at tissue level, the previously assumed abnormally low bone turnover in children with newly diagnosed ALL.

Surprisingly, our qBEI analyses revealed hypomineralization of the bone matrix in the biopsy samples, reflected by a marked decrease in CaMean, CaPeak and concomitantly a nearly 3-times increase in CaLow and elevated CaWidth compared to pediatric reference values [27]. Indeed, normally in situation of low bone turnover, the relative tissue age and consequently the degree of mineralization of the bone matrix increases [20, 35–37]. Low bone turnover associated with low bone matrix mineralization and/or increased heterogeneity in mineralization suggests abnormal osteoblast—and possibly also osteocyte—function, since osteocytes orchestrate both, bone homeostasis and mineralization [38–42].

Noteworthy, BMD *z*-scores were mainly within normal range in our ALL children. A single DXA scan is not very sensitive in showing bone metabolic changes, since there are usually no previous scans available for comparison. We did not find clinically meaningful correlations between BMD *z*-scores and BMDD or histomorphometric parameters. This could be partly due to the late timing of DXA scan and the rather small sample size. Nevertheless, it is plausible that bone microarchitecture and metabolism are distorted before significant changes in BMD can be detected by DXA. Hypomineralization of the matrix indeed makes the bone tissue weak and in combination with low bone turnover predisposes to further decrease in bone volume and fractures. It will be important to determine by following up whether bone matrix hypomineralization identified by qBEI at ALL diagnosis associates with later clinical bone complications in our cohort. Ideally, the BM biopsy studies should be repeated in a longitudinal study setting to evaluate potential cumulative negative effects of cancer treatment on bone quality. Moreover, our ongoing studies will hopefully show whether bone biopsy findings are associated with clinically more feasible parameters of bone health, including circulating bone markers.

Interestingly, recent studies suggest a link between BMD drop during the ALL treatment and osteonecrosis [17, 43]. This association may be due to multiple factors, but one might speculate whether these bone complications share common and cumulative underlying pathomechanisms stemming from very early impairment of bone matrix mineralization, due to dysfunctional osteoblasts and osteocytes.

We could not show a clear association between more advanced bone marrow disease (> 90% by flow cytometry) and bone metabolic or structural parameters. This is probably because in pediatric ALL, bone marrow is typically almost full of blasts in all patients. Moreover, bone marrow aspiration samples may be diluted with peripheral blood and small differences in blast count are therefore not measurable. Instead, it is interesting to speculate whether the length of the disease course before ALL diagnosis might parallel bone deterioration. Thus, long lasting, slow disease progression could lead to greater impairment of bone turnover.

We further showed marked bone marrow fibrosis in half of our ALL patients. Excess reticulin fibrosis particularly in pediatric pre-B-ALL at diagnosis has also been reported earlier [44]. Interestingly, higher levels of BM fibrosis in B-cell ALL patients correlated with poorer response to and overall survival after CAR T treatment. This may be due to attenuated infiltration of CD3 cells into the reticulin rich marrow [45]. Of note, some fibrosis was also seen in our two T ALL samples. The role and prognostic significance of BM fibrosis in ALL remains uncertain.

We recognize some limitations in our study. First, for ethical reasons we did not obtain normal-sized biopsy specimens as recommended for bone histomorphometry [30]. Thus, we could not evaluate structural parameters such as cortical width or trabecular bone volume. Also, dynamic parameters of bone formation were not available since diagnosis cannot be delayed due to tetracycline labelling in a child with suspected ALL. Finally, BMD measurements by DXA were performed within 4 weeks of diagnosis, when leukemia treatment was already started. This is again because it was neither feasible nor ethical to perform DXA scans during ALL induction therapy.

In conclusion, our study revealed a marked suppression in bone turnover and abnormally low and heterogenous bone matrix mineralization in children with ALL already at diagnosis. This may explain why skeletal complications often occur before or at very early stages of ALL treatment. Further studies are needed to address clinical long-term significance of the observed changes.

## Data Availability

The research data on qBEI and histomorphometry supporting this publication can be accessed at our institutional digital data repository for published research via https://creed.lbg.ac.at.
